# Laproscopic Management of Wandering Biliary Ascariasis

**DOI:** 10.1155/2012/561563

**Published:** 2012-08-16

**Authors:** Umesh Jethwani, G. J. Singh, P. Sarangi, Vipul Kandwal

**Affiliations:** ^1^Deparment of Surgery, Vardhman Mahavir Medical College & Safdarjung Hospital, New Delhi 110029, India; ^2^Deen Dayal Upadhyay Hospital, New Delhi, India

## Abstract

Ascariasis is one of the most common helminthic diseases in humans, occurring mostly in countries with low standards of public health and hygiene, thereby making ascariasis highly endemic in developing countries. In endemic areas, 30% of adults and 60–70% of children harbour the adult worm. Biliary ascariasis is a rare cause of obstructive jaundice. Conventional management involves endoscopic extraction of worm. We are reporting a rare case of ascaris which induced extrahepatic biliary obstruction in a young male who presented with acute cholangitis. The ascaris was removed by laparoscopic exploration of the common bile duct. Postoperative period was uneventful.

## 1. Case Report

**Figure 1:**
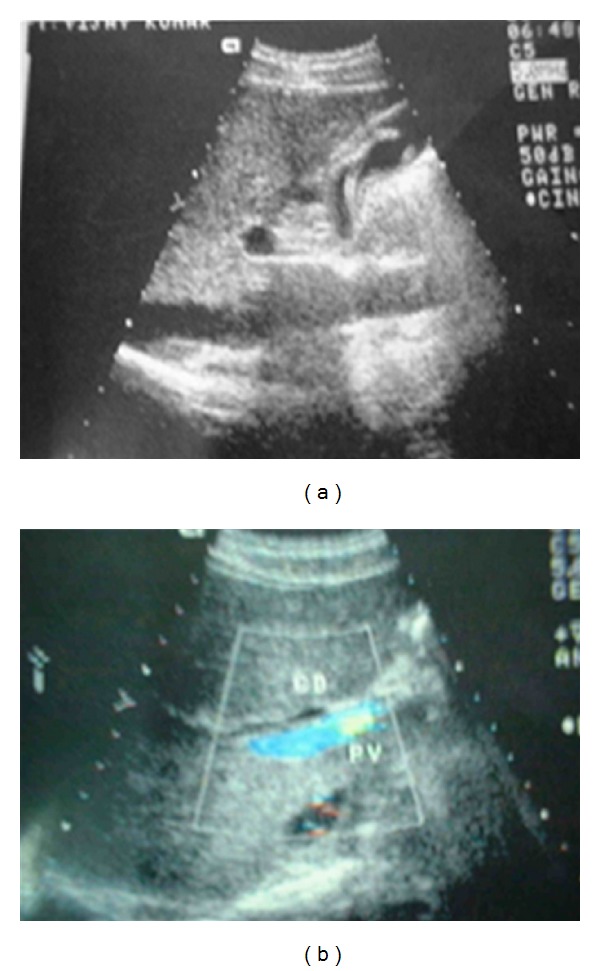
USG—worm in gall bladder and common bile duct.

**Figure 2:**
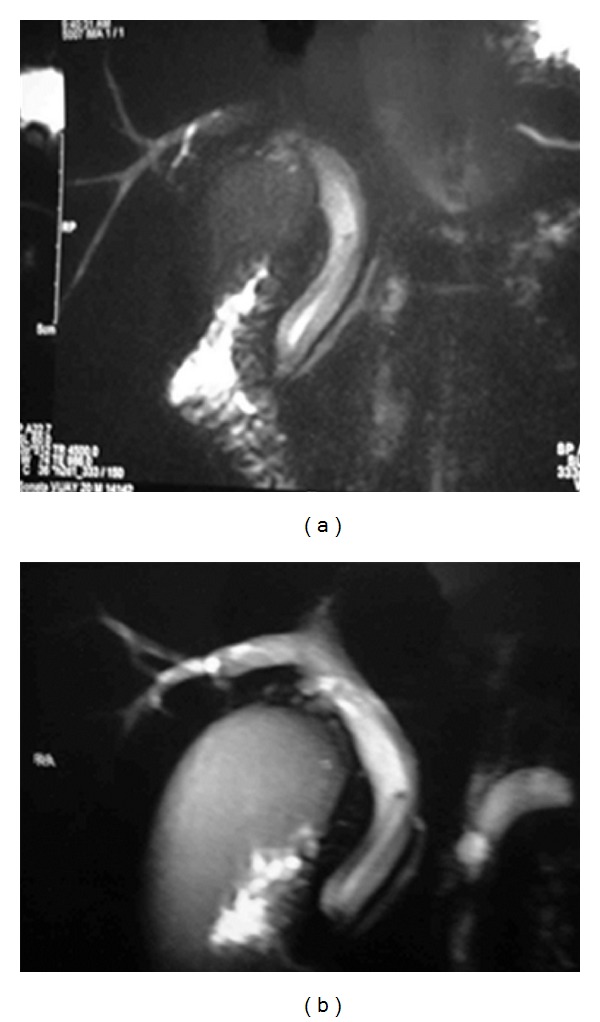
(a) MRCP—hypointense tubular filling defect in CBD and CHD. (b) CBD—9 mm GB—distended, wall thickening and luminal sludge.

**Figure 3:**
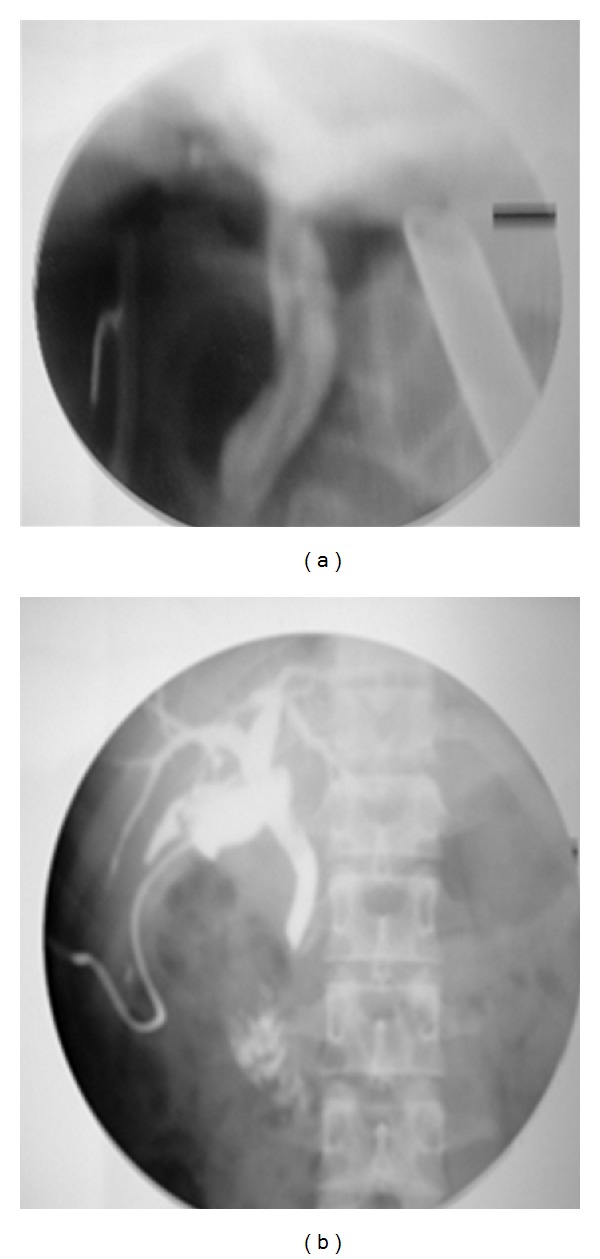
(a) *PerOp cholangiogram* before extraction of worm, (b) after extraction of worm.

**Figure 4:**
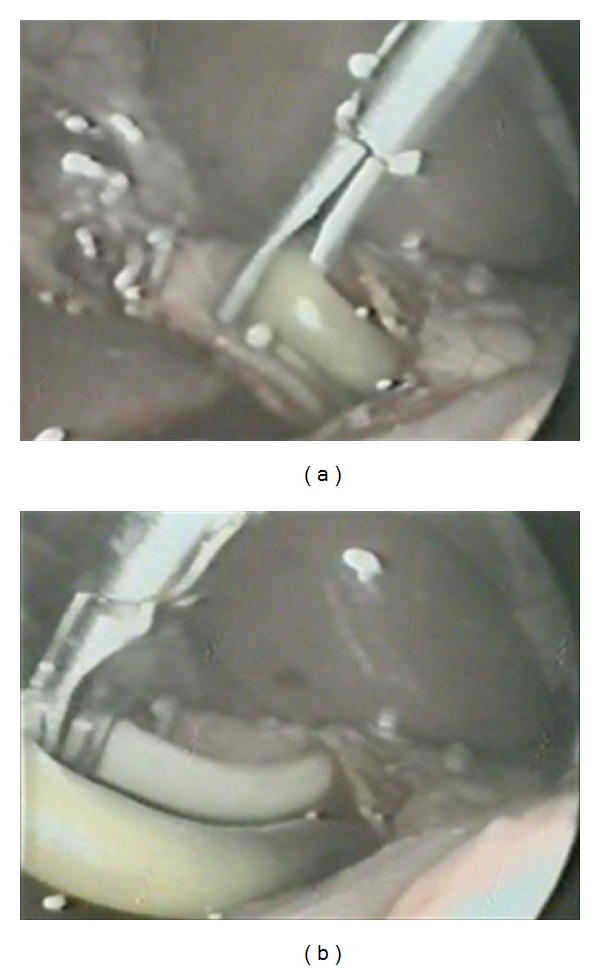
Intraoperative: extraction of common bile duct ascariasis.

**Figure 5:**
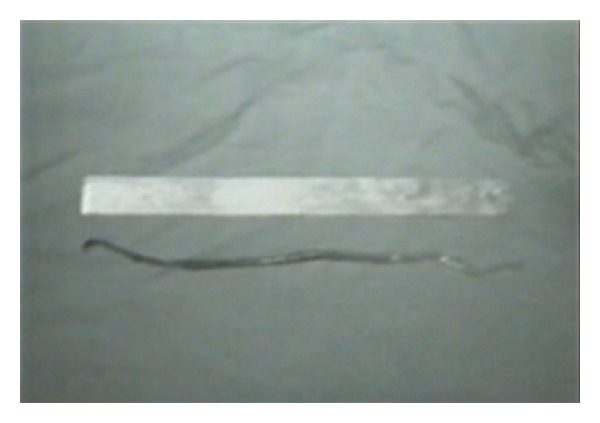
Ascaris worm.

A 20-year young male presented with 3-day history of fever, right upper quadrant abdominal pain, and jaundice. He had no viral syndrome, history of any offending drug intake or any surgical intervention. He had history of passing worms in stool. On examination, he was conscious oriented, hemodynamically stable and had icterus. He had no edema or lymphadenopathy or signs of chronic liver disease. His pulse was 92/minute, body temperature 102°F. His abdominal examination was within normal limits. On evaluation he had leucocytosis (TLC 14,800) with predominant neutrophilia (P86, L8). His liver function test revealed serum bilirubin of 7.2 mg/dL and SGOT and SGPT levels of 48 and 56 (reference range SGOT/SGPT 22/24). He had marked elevation of serum alkaline phosphatase of 55 KA units (reference range 11–13 KA units). His renal function test was normal. His blood culture was sterile. His stool examination was positive for ova of Ascaris. Ultrasound revealed an echogenic linear shadow in the common bile duct and grossly dilated intrahepatic biliary ductal system (Figures 1(a) and 1(b)). His cholangitis was managed by IV fluids, antibiotics, and antihelminths. MRCP was suggestive of hypointense tubular filling defect in common bile duct and common hepatic duct (Figures 2(a) and 2(b)). Despite multiple attempts, worm could not be removed by ERCP. Repeat ultrasound showed worm partly in gall bladder and partly in CBD. Laparoscopic exploration of common bile duct was done and the worm was removed from CBD (Figures 4(a), 4(b) and 5). PerOp cholangiogram was done (Figures 3(a) and 3(b)). T tube was placed in situ. Postoperative period was uneventful. Intermittent clamping of T tube was done from 9th postoperative day and on 14th POD T tube cholangiogram was done which did not show any filling defect in CBD. T tube was removed and patient was discharged after 3 days without any complication.

## 2. Discussion

Ascariasis is a common helminthic disease in developing countries, especially in the tropical and high temperature regions [1, 2]. In the human infection, life cycle begins by ingestion of an egg, with the larvae hatching in the small intestine. The larvae invade small-bowel mucosa, migrate through the circulatory system to the lungs, invade the alveoli, ascend the tracheobronchial tree, and then are swallowed into the small intestine where they mature into adult worms [3]. Intestinal infestation is often asymptomatic, but may cause symptoms such as abdominal pain on right upper quadrant, nausea, vomiting, diarrhea, and loss of appetite, as observed in our patient. Migration of worms into the biliary tree is a well-known complication, which may result in biliary colic, cholecystitis, cholangitis, intrahepatic abscess, or pancreatitis [4, 5]. Dead ascarids can produce chronic inflammatory reaction in the ductal mucosa leading to strictures. Less common complications are necrosis or perforation of common bile duct or perforation of gall bladder. Complications may be fatal and chief cause of death is septicaemia associated with biliary and hepatic complications.

Presentation forms of BA are biliary colic (56%), acute cholangitis (25%), acute cholecystitis (13%), acute pancreatitis (6%), and, rarely, hepatic abscess or haemobilia [1]. Elevation of serum values of bilirubin, transaminases, AP, and GGT can be present, depending on the extent of the biliary obstruction. Leucocytosis can be present and eosinophilia, when present, can give rise to a suspicion of parasitosis. The only specific test, however, is the identification of the parasite in stools or duodenal contents, which can, therefore, be negative. In endemic areas, the diagnosis is based on clinical findings and abdominal US and is confirmed by endoscopic procedures [6]. US shows a typical longitudinal image of a hyperechogenic tubular structure without acoustic shadowing or a transverse image of a round hyperechogenic structure with a hypoechogenic centre [6]. “Strip,” “inner tube,” and “spaghetti” signs have been described [7]. Typical CT findings have also been described, but this technique is helpful especially in the case of liver or pancreatic involvement. However, some times, only indirect, nonspecific signs, such as mild dilatation of the biliary tract can be observed and in developed countries the images seen on US and CT can easily be mistaken for stones or malignancy with consequent delay in diagnosis or inadequate treatment. Magnetic resonance cholangiography can be diagnostic, showing worms as hyperintense tubular structures or intraductal linear filling defects [8, 9]. Real-time percutaneous or endoscopic US can also show the worm moving within the biliary ducts and thereby help to control its position and vitality [10]. ERCP is of fundamental importance in BA, as it can play a diagnostic and therapeutic role, making it possible to view the worms directly and extract them. False negatives are possible due to the movement of the worms, which go in and out of the biliary tree. Sphincterotomy can facilitate remigration of the worms into the biliary tract in endemic areas. For this reason balloon dilatation of Oddi's sphincter is to be preferred to sphincterotomy in these zones. To avoid recurrence, antihelminthic treatment for long periods is required to eradicate the intestinal worms in these areas. On the other hand, the reiterative passages of the worm can make the papilla patulous, making catheterisation easier [6]. Differential diagnosis versus biliary tract lithiasis can be difficult and the two conditions can be associated. In these cases, the risk of treating stones and leaving the worm in place is high, with the consequent need for reiterative procedures [11]. Treatment can be conservative, endoscopic, or surgical. Medical treatment consists in fasting, intravenous fluids, antibiotics, and antispasmodics and aims to push the worms out of the bile ducts. To avoid the death of the worm in the biliary tree, vermifuge must not be given until the symptoms have been resolved. For this reason, albendazole infusion via a nasobiliary drainage, which has been proposed, is contraindicated. Clinical and ultrasonographic monitoring is indicated for 72 hours; then, if symptoms persist, endoscopy is mandatory. If performed by experts, ERCP is successful in 90% of cases, even if multiple sessions may be required. Worms protruding from the papilla can be removed with a grasping forceps; those that are entirely within the bile duct are stimulated to migrate out of the papilla after contrast injection or can be extracted by the Dormia basket or balloon. If the worm is in the pancreatic duct, there is a risk of pancreatitis, and emergency endoscopic treatment is required. Endoscopic failure is the main indication for surgery, together with gallbladder ascariasis, and a few cases of liver abscesses, intrahepatic ascariasis, or severe acute pancreatitis. Surgical procedures include choledochotomy and removal of worms (and calculi), cholecystectomy, and drainage of liver abscesses; usually a T tube is left in the main bile duct. In particular cases, choledochoduodenostomy, hepaticojejunostomy, and pancreaticojejunostomy have also been performed. In the case of ascariasis of one lobe of the liver, hepatectomy is the treatment of choice. Increasing population migration makes it necessary for gastroenterologists, endoscopists, and surgeons worldwide to bear in mind the possibility of this sporadic condition, particularly in patients coming from endemic zones. The diagnosis can be difficult but an accurate diagnosis will help to treat patients promptly and in the most conservative way, as well as to avoid reiterative procedures.

## 3. Conclusion

Ascaris should be a part of the differential diagnosis of common bile duct obstruction. Endoscopic sphincterotomy and bile duct clearance along with pharmacotherapy are the mainstay of treatment. ERCP failure may be due to the presence of ascariasis in the gall bladder and due to stricture or stones. Various options available are surgical exploration and laparoscopic extraction of the worm and biliary stones. However such procedures are usually challenging and require high technical expertise.
